# Compensation for Dental Services

**Published:** 1880-10

**Authors:** E.G. Betty

**Affiliations:** Cincinnati


### COMPENSATION FOR DENTAL SERVICES.

Editor Register:—In the September number of the *Register* appeared an article on the "Relations of the Profession and the Public" from the pen of Dr. Brackett. The paper in the main is a good one, containing many points that are worthy of serious consideration by us all.

We all agree with the Doctor, that dentistry is a noble and liberal profession, that the dentist is or ought to be a man of honor, that he shoulders his share of responsibility in the great system of social economy and that he is called upon to act the part of an educator.

There is one point, however, which the Doctor passes altogether too lightly, and with due respect to him, we crave the liberty of differing and showing wherein he errs. "Do not understand me as clamoring for high fees. Not at all. The dentist who works for one dollar an hour may be just as worthy a man, and just as faithful a practitioner, as he who asks ten." Now then, what are we to understand by these words?

As we see it, all men are *not* born equal, either as regards physical constitution and power or intellectual ability. No matter in what sphere of life a man may engage, he certainly cannot rise to distinction unless he be endowed by nature with the brain to command. As society is now constituted, there are many fields that offer every inducement and opportunity for the employment of intellects of the highest order.

The naval and military departments of the foremost governments are highways leading to great personal distinction, for any one possessing in an eminent degree the force of brain. The law, medicine, fine arts, and many other departments of human knowledge, number amongst their votaries, intellects of the highest type, occasionally a genius. Why is it? Because, in any one of these fields the man of brain finds not only full scope for his powers but an equivalent for his services, rendered in money. Throughout the ranks of the whole army of dentists there is not a single man possessing the genius of a Virchow, a Daniel Webster, or a Meissonier. Just in proportion as a pursuit offers rapid promotion and high office, together with liberal recompense, will it number among its followers individuals of great mental capacity. Dentistry is yet an infant, it cannot afford to employ a high-type brain, it is too poor to pay the piper.

There are no endowments of sufficient wealth connected with any dental school, that can offer to an individual possessing first-class brain, the same amount of recompense that could be obtained in either of the employments named. If the teaching of dentistry will not pay, then its practice must furnish emolument in proportion to the quality of service rendered. 'Tis upon this *quality* that rests our hopes for the future elevated standard of dentistry. If it were impossible to obtain more than one dollar an hour for dental service, five years hence, the dental profession would have ceased to be a noble and liberal calling, merely from the fact that its brains would seek a more profitable market.

Dentistry owes its present status to the few men of brain that have engaged in its study and practice. Were we to shut them out in the future by reducing to a minimum the remuneration such service deserves, the " profession " would gravitate to a very low level. One practitioner may be as worthy and faithful as another, but they do not all possess the same ability. The man of cultured mind and highly skilled fingers can find both employment and pay for his capabilities. The much maligned public is not so great an ass as many dental writers and speakers would have us suppose, and 'tis well that it is so.

Let every individual dentist cultivate both body and mind.

If he cannot vie with the great of the earth, he will at least, not be classed with the incapables and slothful, either by his brethren in particular or the public in general.

" High fees " then are not a bugbear to the advancement of practical dentistry.

In every large community are found many wealthy and intelligent people who are not only able, but willing, to pay for the best class of dental service.

E. G. BETTY, D.D.S.

CINCINNATI, SEPT. 20TH, 1880.

